# Heterozygous *FMN2* missense variant found in a family case of premature ovarian insufficiency

**DOI:** 10.1186/s13048-022-00960-y

**Published:** 2022-02-28

**Authors:** Jie Li, Tianliu Peng, Le Wang, Panpan Long, Ruping Quan, Hangjing Tan, Minghua Zeng, Xue Wu, Junting Yang, Hongmei Xiao, Xiaobo Shi

**Affiliations:** 1 Reproductive Medicine Center, Department of Obstetrics and Gynecology, The Second Xiangya Hospital, Central South University, Changsha, 410011 China; 2 Institute of Reproductive & Stem Cell Engineering, School of Basic Medical Science, Central South University, Changsha, 410013 China

**Keywords:** *FMN2*, DNA damage, DNA repair, POI, Ovarian function, Ovarian reserve

## Abstract

**Background:**

Premature ovarian insufficiency (POI) plagues 1% of women under 40, while quite a few remain an unknown cause. The development of sequencing has helped find pathogenic genes and reveal the relationship between DNA repair and ovarian reserve. Through the exome sequencing, our study targets screening out the possible POI pathogenic gene and variants in a Chinese family and 20 sporadic POI patients, preliminarily exploring the functional impact and finding out potential linkages between the gene and POI.

**Results:**

The whole exome sequencing suggested a novel *FMN2* heterozygous variant c.1949C > T (p.Ser650Leu) carried by all three patients in a Chinese family and another c.1967G > A(p.Arg656His) variant in a sporadic case. Since no *FMN2* missense mutation is reported for causing human POI, we preliminarily assessed p.Ser650Leu variant via cross-species alignment and 3D modeling and found it possibly deleterious. A series of functional evidence was consistent with our hypothesis. We proved the expression of FMN2 in different stages of oocytes and observed a statistical difference of chromosomal breakages between the POI patient carrying p.Arg656His variant and the health control (*p* = 0.0013). Western Blot also suggested a decrease in FMN2 and P21 in the mutant type and an associated increase in H2AX. The p.Arg656His variant with an extremely low frequency also indicated that the gene *FMN2* might play an essential role in the genetic etiology of POI. To the best of our knowledge, this is the first POI report on missense variants of *FMN2*.

**Conclusion:**

This finding indicates a novel gene possibly related to POI and sheds lights on the study of *FMN2*.

**Supplementary Information:**

The online version contains supplementary material available at 10.1186/s13048-022-00960-y.

## Background

Premature ovarian insufficiency (POI), known as premature ovarian failure (POF) before, has been a severe problem that plagues women under 40 due to its damage to fertility and the detrimental effect caused by steroid-deprivation associated symptoms. This disorder is normally diagnosed by (i) amenorrhea for at least 4 months and (ii) an elevated FSH level > 25 IU/l on two occasions > 4 weeks apart [1]. POI could lead to infertility and bring a series of short-term or long-term complications caused by low estrogen levels, which seriously threatens women's health and life quality. Moreover, this is a state of continuous irreversibility. Once the disease is diagnosed, the major treatment is to improve the symptoms consequent on the low estrogen level and reduce complications. For the significant reduction in fertility, donor egg is arguably the only realizable solution. According to statistics in 1986, the total prevalence rate of POF was about 1–2% in Rochester, Minnesota [2, 3]. The prevalence rate of women was 1:1,000 under 30 years of age and 1:10,000 under 20 years of age, which may also have differences in demographic characteristics such as race [4]. However, currently, there is no solid predictive test to identify women who will develop POI unless a mutation known related to POI was detected.

POI is heterogeneous with a broad spectrum of causes, i.e., cytogenetic, genetic, infectious, or iatrogenic. Autoimmune and metabolic etiologies may also be genetic [5]. Besides chromosomal abnormalities, some single-gene mutations unequivocally have a deleterious effect in at least one population. Nevertheless, identifying exact causative genes has been challenging.

In recent years, many studies have directed familial POI etiology toward genes crucial during meiosis, such as generating and repairing Double-strand breaks (DSBs), chromosome synapsis and recombination, and sister chromatid cohesion. Functional studies have proved that mutations in many genes like MCM8 [6], MCM9 [7], STAG3 [8], BRCA2 [9], and CSB-PGBD3 [10] could cause aberrant DNA damage repair. It can then lead to abnormal oocyte morphology or increased number of oocyte apoptosis and thus decrease ovarian reserve. As genome sequencing advances, more candidate genes are to be manifest.

Formin 2 (*FMN2*) is a gene belonging to the formin homology protein family, which is thought to encode proteins essential in the organization of actin cytoskeleton and cell polarity. This protein mediates the formation of an actin mesh that positions the spindle during oogenesis and regulates the formation of actin filaments in the nucleus. Recent papers identified *FMN2* as a novel regulator of the cyclin-dependent kinase inhibitor p21 [11, 12]. They claimed that *FMN2* could enhance the expression of the cell-cycle inhibitor p21, which is a key point to start the DNA damage repair process by preventing its degradation, and *FMN2* itself can be induced by DNA damage as well. These reports corroborate *FMN2*’s potentially significant role in DNA repair. However, whether *FMN2* can be a potential gene related to POI or not still asks for in-depth investigation.

Through the exome sequencing of family POI and sporadic POI, our study targets three aspects of research:

1. Screen out the possible pathogenic gene and mutation in this family.
2. Preliminarily explore the functional impact of the mutation in *FMN2*.
3. Find out potential linkages between the pathogenic gene *FMN2* and POI.

### Case report

**Fig. 1:**
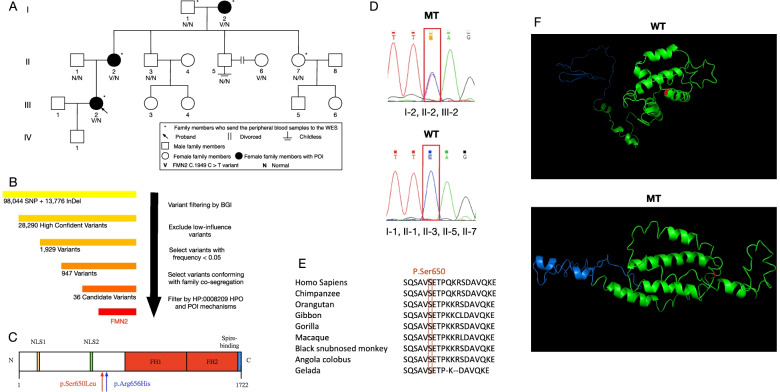
Screening and preliminary prediction of the candidate gene. **A** Pedigree of the index family. **B** Flowchart of the variant filtering process. **C** Simplified structure chart of human protein *FMN2* (1722aa). N = N-terminus, C = C-terminal tail. Major functional regions FH1, FH2 are colored in red, nuclear localization sequence 1 and 2 (NLS1, NLS2) are colored in orange and green respectively, spire-binding regions are marked with blue, and the black arrow points to the mutation sites. **D** Sanger sequencing results of p.650 in the family case. **E** Cross-species alignment of *FMN2*. □ denotes p.650 site in *FMN2* proteins, which is serine in 9 primates. **F** Simplified 3D Cartoon models for human *FMN2* protein. The mutant sites were colored in red, FH2 domains were colored in green, while other residues were colored in blue. MT mutant type; WT wild type

Three members of a three-generation nonconsanguineous Han Chinese family presented with secondary amenorrhea before their age of 40 (Fig. 1A). They are the proband (Fig. 1A III2), the proband’s mother (II2), and proband’s grandmother (I2). The proband in this family was a 34-year-old woman who presented with oligomenorrhea and rapidly progressed to menopause two years after she gave birth to her only child in 2017, without a history of any surgery before. Her FSH level was 26.44 IU/L, and diagnosed as POI several months later, with basal FSH level exceeding 25 IU/L twice and a significantly smaller uterus and ovarian size in pelvic ultrasound but a normal female karyotype (46, XX). She is now treated with estrogen and progesterone replacement therapy and experiences regular menstrual cycles. Another two affected members, I2, II2 presented with amenorrhea at the age of 37 and 40, respectively. Besides, I2 was diagnosed with advanced breast cancer in 2018, at the age of 77, details about which remain unknown (See Table 1 for more clinical information). The proband’s younger uncle (II5) divorced in his early year of marriage before he can have a child and has never remarried since then. No other family history of anemia, blood dyscrasias, photosensitivity, or immunodeficiency has been found. The proband’s 50-year-old aunt (II7) and her three young female cousins under 40 (III3, III4, III6) are still under normal menstrual cycles, and we will keep tracking.

**Table 1:** Clinical features of POI patients with mutations in FMN2

Patient No	FMN2 Mutations			Current Age (yr)	Age of Amenorrhea (yr)	Smoking status	No. of pregnancies	Concomitant diseases
	**Sequence Variation**	**Amino-acid Variation**	**Mutation Type**					
**Index Family**								
l**2**	c.1949C > T	p.Ser650Leu	SNV	81	37	None	4	Breast Cancer, Hypertension
ll**2**	c.1949C > T	p.Ser650Leu	SNV	56	40	None	2	Hypertension
lll**2**	c.1949C > T	p.Ser650Leu	SNV	35	34	None	1	None
**Sporadic POI**								
**Case 1**	c.1967G > A	p.Arg656His	SNV	29	23	None	0	None

Peripheral-blood samples were obtained from three affected (I2, II2, III2) and five unaffected (I1, II1, II3, II5, II7) family members. All members mentioned above provided their written informed consent to participate in this study and agreed with the publication of this case report.

## Results

### Identification of *FMN2* as candidate gene

Based on the filtering standards mentioned in Fig. 1B, we filtered candidate variants from the total variants obtained from the WES report. Comparing with HP:0,008,209 HPO, which recorded all genes reported to be pathogenic in POI, no known genes were found in this family report. We then considered other genes with mechanisms highly relevant to ovarian development (reproductive endocrine, follicle development, meiosis and DNA repair) among the rest 36 candidates. Finally, the variant ENST00000319653:c.1949C > T in *FMN2* leading to a missense from serine to leucine (p.Ser650Leu) in human *FMN2* protein is targeted. This variant is heterozygous and has never been reported in any database before. Hence, the impact that the mutation brought to this protein remains unknown. Considering that this variant is novel, most of the in-silico tools are not applicable due to their restrictions in information types and scopes. We analyzed this variant through a reliable in-silico tool, i.e., Combined Annotation Dependent Depletion (CADD), which integrates multiple annotations into one metric. A score of 13.390 was yielded indicating possibly deleterious. As is shown in the simplified protein structure (Fig. 1C), the human *FMN2* gene encodes a 1722-aa *FMN2* protein reportedly [13]. FH1 domain (codon 810–1268) and FH2 domain (codon 1283–1673) are the binding regions for α-catenin, two putative NLS (nuclear localization sequence) regions are located near the N-terminus of *FMN2* [14], beginning at 6 and 411, respectively. And the spire-binding domain is reported to be the C-terminal tail of *FMN2* (1706–1722) [15]. Sanger sequencing was applied in all eight blood samples to verify its existence in three family members with POI. As shown in Fig. 1D, healthy relatives were wild homozygotes, while POI patients carried a C > T heterozygous variant.

We obtained *FMN2* amino-acid sequences of different species from a genomic browser in Ensembl to analyze the evolutionary conservation of this mutation and found it highly conservative in all primates (Fig. 1E). There is no report regarding this mutation in the population before, and the total sequence of *FMN2* protein is extremely large (1722aa). Hence, there is no existing protein model for comparison. We constructed cartoon 3D protein models based on the most harmful transcript of *FMN2* (ENST00000447095, 228aa), which has an FH2 domain. Our mutation can cause a p.Ser87Leu missense in this transcript. Figure 1F depicts the cartoon 3D prediction models of *FMN2* protein and the mutant type, which shows that the mutation might cause huge structural changes. The helix of the mutant region changes into chains. The 3D structure of the FH2 domain changes into a completely different one, accompanied by a decrease in hydrophilicity, which is important for the function.

### *FMN2* was expressed in germ cells of human fetus ovary

**Fig. 2:**
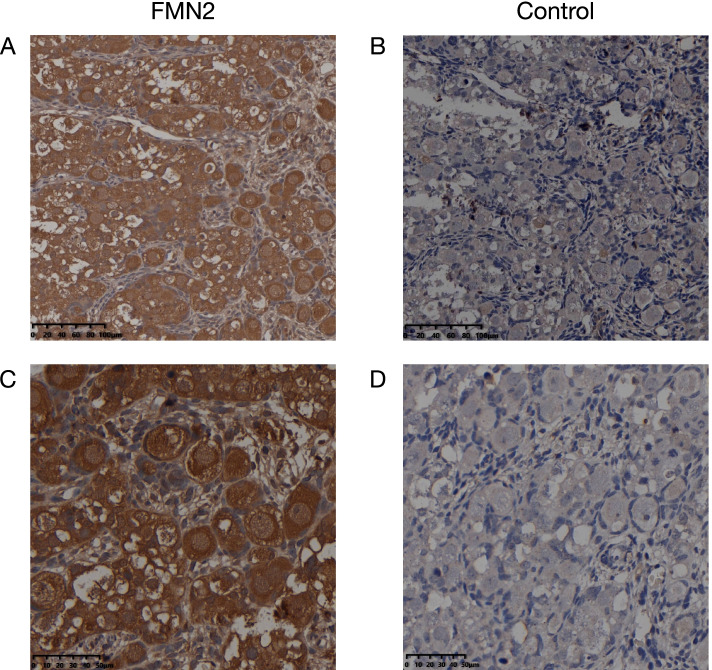
*FMN2* expression in 25w human fetus ovary. Scale bars, 100 μm and 50 μm

Immunohistochemical studies in the 25-week human fetus ovary show that *FMN2* protein expresses in both the nuclei and the cytoplasm of primordial follicles, antral follicles, and other oocytes in different development stages (Fig. 2). Staining is also shown in follicular fluid and intercellular substance but not in any granulosa cell. It suggests that there is a basis for protein *FMN2* to function since the early stage of follicle development.

### The *FMN2* mutation caused reduced function in DNA damage repair

**Fig. 3:**
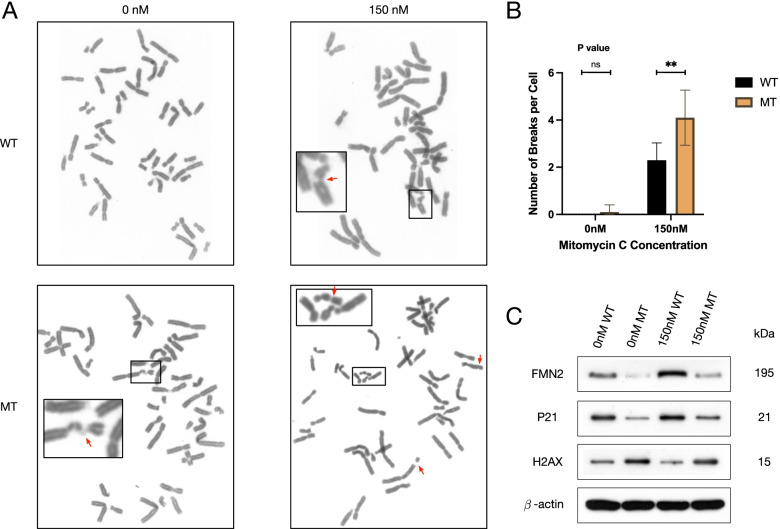
DNA damage models induced by MMC. **A** Chromosomal breakages in the peripheral lymphocytes obtained from the proband (III-1) and an unrelated control. ↖ denotes the chromosome break point. **B** Average number of chromosomal breaks in different MMC concentrations. Number of * denotes the degree of difference; ns means nonsense. **C** Expression of *FMN2*, P21, and H2AX in different MMC concentrations. performed by Western blot. WT wild type; MT mutant type

To verify the difference in the DNA repair between the mutant and wild types of *FMN2*, we observed the chromosomal morphology of lymphocytes (Fig. 3A) and counted the number of chromosomal breaks per cell in different concentration of MMC (20 cells per sample per MMC concentration). According to our statistics (Fig. 3B), the average number of chromosomal breaks in the mutant type was larger than that of the wild type in each concentration of MMC, and the difference under 150 nM MMC was statistically significant (*p* = 0.0013). However, there were too many necrotic cells in both the mutant type and the wild type under the 300 nM condition, and it was difficult to observe the breaking point or even enough karyotype.

### The *FMN2* mutation led to lower protein expression in DNA damage model

H2AX is a key factor in DNA damage repair [16], and it is commonly applied as a biomarker assessing other related DNA damage proteins. We isolated lymphocytes and extracted protein treated with 150 nM MMC in the same way described above. Western Blot was applied to compare protein expression differences between the wild type and mutant type before and after the MMC treatment (Fig. 3C). Quantitative analyses of gray values are shown in Supplementary Fig. 1. As shown in our results, *FMN2* expressed lower in the mutant type (the proband) than the wild type (unrelated control), and both showed higher expression under 150 nM MMC. This indicates that p.Ser650Leu missense may lead to haploinsufficiency in *FMN2* expression. P21 showed rather lower expression in the mutant type, and it had an increased expression under MMC treatment. H2AX expression was rather higher in the mutant type, and it reduced while treated with MMC.

### Another *FMN2* variant was discovered in sporadic POI cases

After obtaining the WES results of 20 sporadic POI patients, we scanned all the variants and found another heterozygous *FMN2* variant ENST00000319653.9:c.1967G > A (p.Arg656His) in one of the patients (Fig. 1C, Table 1). This is a variant with an extremely low mutation frequency (0.0000577 in ExAC), and it gets a prediction as possibly deleterious in 3 kinds of different in silico tools (Table 2). Thus, rare variants carried by family and sporadic POI patients reflect the possible role of the *FMN2* gene in POI pathogenesis.

**Table 2:** p.Arg656His Variant Prediction by in silico Tools

Software	Score	Prediction
Polyphen-2	0.733	Possibly damaging
M-CAP	0.050	Possibly pathogenic
CADD	12.700	Possibly deleterious

## Discussion

In this paper, we found a novel *FMN2* heterozygous variant c.1949C > T (p.Ser650Leu) carried by all three patients in a Chinese family, and another *FMN2* heterozygous variant c.1967G > A(p.Arg656His) in a sporadic case, through the whole exome sequencing. Since no *FMN2* missense mutation is reported for causing human POI, we preliminarily assessed p.Ser650Leu variant via cross-species alignment and 3D modeling and found it possibly deleterious. A series of functional evidence was also consistent with our hypothesis. We proved the expression of FMN2 in different stage of human oocytes, and observed a statistical difference of chromosomal breakages between the POI patient carrying p.Arg656His variant and the health control (*p* = 0.0013). Western Blot also suggested a decrease in FMN2 and P21 in the mutant type, and an associated increase in H2AX. The p.Arg656His variant filtered from 20 sporadic cases with an extremely low variant frequency also indicated that the gene *FMN2* might play an essential role in the genetic etiology of POI. To the best of the authors’ knowledge, this is the first report on missense variants of *FMN2* that cause POI.

### POI and DNA damage repair

Premature Ovarian Insufficiency has been a severe problem for female reproductive health. As a heterogenous and irreversible disease, many new techniques have been used to achieve the purpose of early diagnosis. About 5% of POI patients have a clear family history of early menopause, suggesting that familial aggregation exists. However, most POI patients still have no clear etiology.

Major causes of POI are follicular dysfunction and premature depletion of functional primordial follicles [17]. The eukaryotic cell has a certain ability of self-repair. If the damage is severe enough beyond repair, the apoptosis mechanism may be activated to remove permanently damaged cells, leading to a decrease in the number of normal cells [18]. For the follicle reserve is usually limited, the function of DNA repair seems far more critical. Before mammals were born, oocytes have been long-term arrested at the first meiotic prophase [19]. A large number of DNA replicates during meiosis will be completed during the same period. Therefore, to ensure the correct replication of genetic information in meiosis I and the proper separation of homologous chromosomes, oocytes have a mechanism of checkpoints at the pachytene stage of meiosis prophase [20]. When DNA damage occurs, checkpoints are activated to arrest the cell cycle and initiate the downstream DNA repair pathway. DNA can be quickly repaired during this time if the damage is minor or there is a perfect DNA repair system. Otherwise, abnormal repair may be formed, leading to cell apoptosis or even tumorigenesis [21–24]. In our cases, in addition to a three-generation POI family, the proband’s grandmother was diagnosed with mammary cancer as well, giving her first and second-degree relatives a much higher rate of morbidity than normal [25, 26].

Mammalian oocytes are particularly vulnerable to DNA damage. Physiologically, they may lie dormant in the ovary for many years (> 40 in humans) until they receive the stimulus to grow and acquire the competence to become fertilized. The implication of this is that in some organisms, such as human, oocytes face the danger of cumulative genetic damage for decades. Given the critical role of meiosis in germ-cell survival, endocrine and fertility problems often occur in persons with a defect in DNA repair [18]. In our lymphocyte models, we found a slightly higher number of chromosomal breaks in the mutant type in each concentration of MMC. And under the condition of 150 nM, the number of breaks between the two groups showed a statistically significant difference, consistent with other MMC induced models [27–30]. Besides, the higher concentration of MMC we used, the more necrotic cells exist. And at 300 nM condition, it was unprecedentedly challenging to find karyotypes and count the breaks because of the full view of necrotizing cells under a microscope. However, we did not find a suitable statistical method to measure the apoptotic cells in our slides, which may cause inaccuracy in the comparison results between the mutant type and wild type. Further functional studies and larger sample size are needed in the future work.

### WES and POI

The theory goes hand in hand with reports of POI cases caused by DNA repair-related genetic abnormalities [31]. Through whole-exome sequencing, Qin et al. [10] discovered three novel mutations in CSB-PGBD3, which caused a dysfunctional DNA repair mechanism. Novel mutations in the gene BRCA2 [9] and FANCA [32] related to DNA repair were also found in the same way.

We have been collecting basic information and peripheral blood samples of POI patients in the Second Xiangya Hospital of Central South University since 2018. In our past work, familial and sporadic POI samples were counted.

In this study, WES and Sanger sequencing are performed in a non-syndromic Chinese POI family, identified the c.1949C > T variant in *FMN2*. Cross-species alignment and 3D modeling predicted it as possibly deleterious. Functional studies further implied the hypersensitivity it caused towards mitomycin C. Generally, these results suggest that the novel mutation leads to lower expression in protein *FMN2*, caused a deficiency in DNA repair. P21 also shows a lower expression, referring to a shorter cell cycle arrest and time for those damage sites to be fixed. Among 20 sporadic POI cases, one additional missense mutation ENST00000319653.9:c.1967G > A (p.Arg656His) in *FMN2* was found simultaneously, which can be a lateral confirmation of the essentiality of *FMN2* on DNA repairing. As a candidate gene causing POI, it needs to be intact for normal ovarian development and maintenance.

WES has helped us discover many candidate genes related to DNA repair in POI pedigrees and sporadic POI cases. However, it is still in the initial phase revealing the genetic etiology of this disease.

### *FMN2* and POI

Formin homology proteins are actin regulators with scaffold function (implicated in organogenesis), normal tissue homeostasis, and invasion and metastasis of cancer cells through the regulation of actin remodeling. Human *FMN2* (1722 aa) showed 74.7% total-amino-acid identity with mouse *Fmn2* and 31.9% total-amino-acid identity with human FMN1. Although the N-terminal half was divergent between *FMN2* orthologs and FMN1 orthologs, FH1 and FH2 domains were conserved among *FMN2* and FMN1 orthologs [13]. Little work has been done on human *FMN2*, but mouse and drosophila *FMN2* homologs are relatively well studied. FMNL1, FMNL2, FMNL3, DIAPH1, DIAPH2, DIAPH3, DAAM1, DAAM2, *Fmn2*, FHOD1, FHOD3, GRID2IP, and FHDC1 are Formin homology proteins with FH1 and FH2 domains [33–36]. With multiple proline-rich motifs, the FH1 domain is the binding region for Profilin, SRC, EMS1, FNBP1, FNBP2, FNBP3, FNBP4, and WBP4 [37–41], while the FH2 domain is the actin-structure modification region [42, 43]. Recent studies declared that mammalian Spir1 and Spir2 KIND domains were reported to bind directly to the C-terminal tail, distal to the FH2 domains, of *Fmn1* and *Fmn2* [44]. The binding domain (residues 1023–1059), which formed Spir1/*Fmn2* Complex with Spir1, are necessary and sufficient to stabilize actin [15]. A study claimed that the nuclear actin assembled by Formin-2 and Spire-1/Spire-2 plays an important role in the DNA repairing procedure, which implies another role of *Fmn2* [14].

However, the function of the rest residues remains unknown. Our work predicted that p.Ser650Leu missense mutation might lead to a tremendous change in the 3D structure of the FH2 domain, which may affect or even lose the function of actin binding. *FMN2* is now reported as a critical regulator of the cyclin-dependent kinase inhibitor p21 [11]. Some existing research claimed that increasing *FMN2* protein levels promotes cell cycle arrest by inhibiting the degradation of p21, which is closely related to the procedure of DNA damage repair [18], verifying the conjecture above in another hypothesis. This phenomenon is highly consistent with our experimental results and provides evidence for the pathogenicity of *FMN2* we identified in this pedigree. Our work proves a lower *FMN2* and P21 expression under MMC treatment, suggesting that when under severe environmental stimuli, DNA damage repair increases, *FMN2* expression increases, and P21 increases accordingly, consistent with previous research. And according to our results, p.Ser650Leu heterozygous variant may lead to a decrease in *FMN2* and p21 expression under the same conditions. H2AX is the first step in recruiting and localizing DNA damage proteins. Former studies in DNA damage models showed a dose-dependent decrease in H2AX when treated with radiation or chemotherapy drugs [45]. In our study, H2AX was decreased in both types under 150 nM MMC, which means more H2AX consumption was needed for DNA repair, suggesting a more serious DNA damage and a DNA repair that is difficult to keep up with on time. Besides, there was a comparatively higher H2AX expression in the mutant type in each condition, indicating a higher DNA damage rate. To all appearances, this heterozygous missense mutation in *FMN2* caused more damage and a shorter repairing time.

*FMN2* has been proved as a significant gene related to mouse reproduction. In a mouse model knocked out 433aa of the FH1 domain, female *FMN2*^−/−^ mice shown a phenotype with abnormal oocyte morphology, abnormal polar body morphology, abnormal female meiosis, abnormal pregnancy, and reduced female fertility [46]. The pathogenicity of *FMN2* in human may be slightly different. According to THE HUMAN PROTEIN ATLAS (https://www.proteinatlas.org/ENSG00000155816-FMN2/tissue), human *FMN2* protein is expressed in brain, ovary, testis, and other tissues. Previous research reported mental retardation cases with *FMN2* nonsense or truncation mutations on different sites [45, 47]. In 2016, another study found a significantly higher copy number variation (CNV) in the *FMN2* gene while statistically comparing the sporadic POF patient population with healthy controls. However, they did not provide a more functional experimental basis [48]. Yet, no *FMN2* missense has been found underlying any POI case. Our work proves the existence of *FMN2* in different stages of human oocytes on protein levels, consistent with the Human Protein Atlas. WES reports of a family POI case and 20 sporadic POI cases provided the novel missense variant p.Ser650Leu, on which we mainly focused in this work, and another rare variant p.Arg656His of *FMN2*, which was predicted to be possibly deleterious by three in-silico tools. Although our sample size is too small to clarify the impact of these two mutations, it is suggested to deem *FMN2* as a candidate gene responsible for POI, acting through insufficient DNA repairing.

## Conclusions

In summary, to the best of the authors’ knowledge, this is the first paper that attempts to discover the novel missense p.Ser650Leu mutation in *FMN2* and report missense variants of *FMN2* related to POI through abnormal DNA repair. Our findings extend the mutational spectrums of *FMN2* and enlarge the candidate gene list of POI, which shows important implications for genetic counseling of patients with POI. However, to confirm the pathogenicity of these variants and the relationship between *FMN2* and ovarian dysgenesis in humans, further study with large samples size is requisite. Assays for chromosomal breaks, particularly, are useful in evaluating patients who present with ovarian dysgenesis. They may indicate that, in some patients, defects in DNA repair are the underlying genetic basis for gonadal dysgenesis. These patients have a predisposition for cancer, which warrants surveillance.

## Methods

### WES, variant filtering, and sanger sequencing

We adopted the TaKaRa MiniBEST Whole Blood Genomic DNA Extraction Kit to extract Genomic DNA (gDNA) from all the peripheral-blood samples. The gDNA samples of three known affected cases I2, II 2, III 2, and two healthy family members II, II 7 were sent to the WES program of BGI for Whole-exome sequencing (BGI Genomics, BGI-SHENZHEN). Total clean reads per sample were aligned to the human reference genome (GRCh37/HG19) using Burrows-Wheeler Aligner (BWA). On average, 99.93% successfully mapped. To secure the accurate variant, we employed the Genome Analysis Toolkit (GATK, https://www.broadinstitute.org/gatk/guide/best-practices) for variant analysis according to the recommended Best Practices. Local realignment around InDels and base quality score recalibration were performed using GATK, with duplicate reads removed by Picard tools. The sequencing depth and coverage for each individual were calculated based on the alignments. In addition, the strict data analysis quality control system (QC) in the whole pipeline was built to guarantee qualified sequencing data. The mean sequencing depth on target regions was 143.10-fold. On average per sequencing individual, 99.74% of targeted bases were covered by at least 1 × coverage, and 99.07% of the targeted bases had at least 10 × coverage. Each sample’s clean reads had high Q20 (over 96.82%) and Q30 (over 89.25%), which showed high sequencing quality (More details of data quality control can be found in Supplementary Table 1, 2, 3).

All candidate variants are required to meet the following criteria:

(i) Variants with low impact (mutations on 3-prime, 5-prime, intron, or non-coding transcript exon, and synonymous mutation) were ruled out.
(ii) Be absent or occurring at a frequency less than or equal to 0.05 in all the following databases: KG, ExAC, ESP, PVFD, CG, HapMap, Wellderly, and three IN-HOUSE databases of BGI (IN-HOUSE-1, IN-HOUSE-2, IN-HOUSE-3).
(iii) Conform to the principle of genetic co-segregation.

Among the rest candidates, variants on genes recorded by the Human Phenotype Oncology (HPO) (HP:0,008,209 https://hpo.jax.org/app/browse/term/HP:0008209) or genes closely related to ovarian function were first considered.

Sanger sequencing was performed in all eight gDNA samples described above and a normal woman gDNA sample not related to the family to verify the mutation of the candidate gene *FMN2*. In addition, specific PCR primers targeting the variant (*FMN2*-F: 5’-TTGGTGAAGTGGCTTCTATCGT-3’ and *FMN2*-R: 5’-CATCTCGCAGACTTCTCCCTG-3’) were designed to amplify the target region. We identified the PCR products by 2.0% agarose gel electrophoresis and sequenced them on ABI 3730 automated sequencer (Applied Biosystems, Forster City, California, USA).

### Evolutionary conservation analysis and bioinformatics analysis

Amino acid sequences of *FMN2* protein in different species were obtained from the Ensembl to analyze the evolutionary conservation. Furthermore, we performed an alignment of different species to evaluate the conservation of the mutant site. For further predictions on the mutation, 3D structure were modeled by I-TASSER (https://zhanglab.ccmb.med.umich.edu/I-TASSER/) and visualized by Pymol (http://www.pymol.org/). For initial evaluation on the p.Arg656His mutation, different types of in silico tools were used as below: Polyphen2 (http://genetics.bwh.harvard.edu/pph2/), M-CAP (http://bejerano.stanford.edu/MCAP/), and CADD (https://cadd.gs.washington.edu/snv/).

### Immunohistochemistry

We used the ovarian tissue obtained from a 25-week human fetus to validate the *FMN2* protein expression in human ovaries. Samples were obtained from the Department of Obstetrics and Gynecology, Second Xiangya Hospital of Central South University, with the Ethics Committee of Central South University’s approval. The tissue sections with embedded paraffin were first dewaxed using the xylene for 20 min and then hydrated by gradient dilution of absolute ethanol. After completely inactivated endogenous peroxidase by covering 3% H2O2 at 37 °C for 20 min, the sections were immersed in 10 mmol/L citric acid buffer with repeated boiling and cooling for antigen retrieval. Afterward, goat serum was used to block for 20 min. The sections were incubated with 1:500 anti-*FMN2* antibody (Proteinteach, Rabbit Polyclonal) at 4 °C overnight and incubated with biotinylated secondary antibody (Goat-anti-Rabbit) at 37 °C for 1.5 h. Diaminobenzidine (DAB) and hematoxylin were then used to stain the sections and mounted with coverslips. Antibody dilutions were used as the negative controls.

### Chromosomal breakage counting and western blot

Mitomycin C (MMC) is a DNA crosslinker that can effectively inhibit DNA synthesis, induce DNA damage, and cause cell apoptosis at high concentrations. It is often used in constructing DNA damage models. We cultured peripheral blood of proband and healthy women of the same age for 72 h and added 0, 150, 300 nM MMC (ApexBio A4452), respectively, 24 h before lymphocytes collecting. After the DNA damage model being built, lymphocytes were extracted. We measured chromosomal breakages in each karyotype (20 karyotypes per sample per concentration) and compared the expression of *FMN2*, p21, and H2AX by Western-Blot.

Lymphocytes were resuspended and lysed in 200 μl Radio Immunoprecipitation Assay (RIPA) buffer with 1 mM Phenylmethanesulfonyl fluoride (PMSF) in Western Blot. After being centrifuged at 12,000 rpm at 4 °C for 15 min, the supernatants were collected, and protein concentrations were measured by the BCA method. Equivalent amounts of protein were separated by 10% SDS-PAGE gel and then electro-transferred to PVDF membranes. We blocked the PVDF membranes with 5% nonfat milk in TBST for 2 h at room temperature and then incubated with primary antibodies overnight at 4 °C. The following primary antibodies are selected: *FMN2* Rabbit Polyclonal Antibody (Proteintech 11,259–1-AP, 1:750), P21 (Proteintech 10,355–1-AP, 1:1000), H2AX (Proteintech 10,856–1-AP, 1:750), β-Actin (Proteintech 66,009–1-Ig, 1:5000). Membranes were incubated with HRP-conjugated anti-rabbit or anti-mouse secondary antibody for 1.5 h at room temperature and subjected to chemiluminescent detection with ChemiDoc MP System (BioRad).

### Statistical analysis

Statistical analyses were performed in the Graphpad prism 8.4.2 software to analyze the variance of two factors. After cleaning the data by Geisser-Greenhouse, we used Sidak's multiple comparison tests to differentiate between groups and drew the histogram. The results were represented with mean ± standard errors, set *p* < 0.05 as significant difference threshold.

## Supplementary Information

**Additional file 1:** **Supplementary Figure 1.** Quantitative analysis of gray values. **Supplementary Table 1.** Summary of WES Data and Data Quality Control. **Supplementary Table 2.** Summary Statistics for SNPs. **Supplementary Table 3.** Summary Statistics for InDels.

## Data Availability

The data supporting the findings of this study is available on request from the corresponding author. The data is not publicly available due to privacy or ethical restrictions.
