# Supplementary material for: Heterozygous FMN2 missense variant found in a family case of premature ovarian insufficiency
Source: J Ovarian Res. 2022 Feb 28;15:31. doi: 10.1186/s13048-022-00960-y (PMC8886936; doi:10.1186/s13048-022-00960-y)
Supplement: Supplementary file 1 — Additional file 1: Supplementary Figure 1. Quantitative analysis of gray values. Supplementary Table 1. Summary of WES Data and Data Quality Control. Supplementary Table 2. Summary Statistics for SNPs. Supplementary Table 3. Summary Statistics for InDels. [file 13048_2022_960_MOESM1_ESM.doc]

## SUPPLEMENTARY APPENDIX

**Heterozygous *FMN2* missense variant found in a family case of premature ovarian insufficiency**

## Jie Li, Tianliu Peng, Le Wang, Panpan Long, Ruping Quan, Hangjing Tan, Minghua Zeng, Xue Wu, Junting Yang, Hongmei Xiao, Xiaobo Shi.

## LIST OF CONTENTS

[SUPPLEMENTARY APPENDIX 1](#__RefHeading___Toc80306354)

[Jie Li, Tianliu Peng, Le Wang, Panpan Long, Ruping Quan, Hangjing Tan, Minghua Zeng, Xue Wu, Junting Yang, Hongmei Xiao, Xiaobo Shi. 1](#__RefHeading___Toc80306355)

[LIST OF CONTENTS 1](#__RefHeading___Toc80306356)

[SUPPLEMENTARY FIGURES 1](#__RefHeading___Toc80306357)

[Supplementary Figure 1. Quantitative analysis of gray values 1](#__RefHeading___Toc80306358)

[SUPPLEMENTARY TABLES 2](#__RefHeading___Toc80306359)

[Supplementary Table 1. Summary of WES Data and Data Quality Control 2](#__RefHeading___Toc80306360)

[Supplementary Table 2. Summary Statistics for SNPs 3](#__RefHeading___Toc80306361)

[Supplementary Table 3. Summary Statistics for InDels 3](#__RefHeading___Toc80306362)

## SUPPLEMENTARY FIGURES

### Supplementary Figure 1. Quantitative analysis of gray values

**
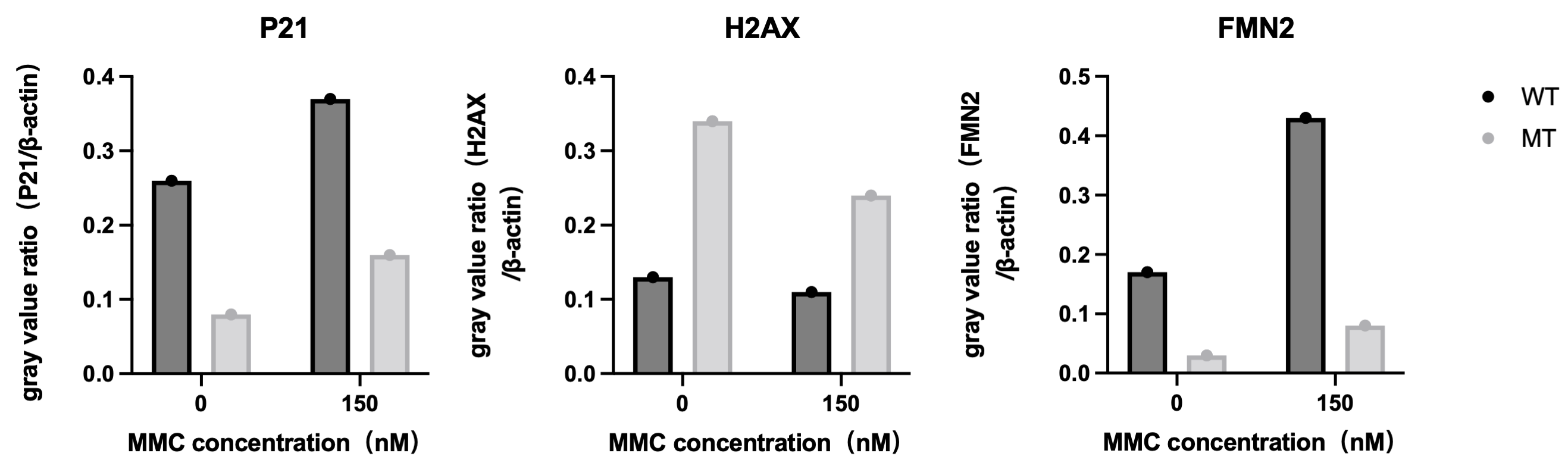
**

## SUPPLEMENTARY TABLES

### Supplementary Table 1. Summary of WES Data and Data Quality Control

| **Sample ID** | **I-2** | **III-2** | **II-2** | **II-7** | **I-1** | **Average** |
| --- | --- | --- | --- | --- | --- | --- |
| Initial bases on target | 60456963 | 60456963 | 60456963 | 60456963 | 60456963 | 60456963 |
| Total effective reads | 118978000 | 151285453 | 120507674 | 122206722 | 124516034 | 127498776 |
| Total effective bases (Mb) | 11851.7 | 15048.4 | 12003.4 | 12124.3 | 12390 | 12683.56 |
| Effective sequences on target (Mb) | 8837.96 | 9077 | 8748.9 | 7668.51 | 8925.19 | 8651.51 |
| Capture specificity (%) | 74.57 | 60.32 | 72.89 | 63.25 | 72.04 | 68.61 |
| Mapping rate on genome (%) | 99.93 | 99.93 | 99.93 | 99.91 | 99.93 | 99.93 |
| Duplicate rate on genome (%) | 12.57 | 11.91 | 11.03 | 9.92 | 10.18 | 11.12 |
| Mismatch rate in target region (%) | 0.33 | 0.32 | 0.35 | 0.34 | 0.33 | 0.33 |
| Average sequencing depth on target | 146.19 | 150.14 | 144.71 | 126.84 | 147.63 | 143.1 |
| Fraction of target covered >= 1x (%) | 99.7 | 99.71 | 99.7 | 99.69 | 99.92 | 99.74 |
| Fraction of target covered >= 4x (%) | 99.55 | 99.59 | 99.54 | 99.5 | 99.77 | 99.59 |
| Fraction of target covered >= 10x (%) | 99.06 | 99.18 | 99.05 | 98.82 | 99.24 | 99.07 |
| Fraction of target covered >= 20x (%) | 97.6 | 97.91 | 97.56 | 96.69 | 97.6 | 97.47 |
| Raw reads | 136122964 | 171768346 | 135492164 | 135696946 | 138658036 | 143547691 |
| Raw bases (Mb) | 13612.3 | 17176.83 | 13549.22 | 13569.69 | 13865.8 | 14354.77 |
| Clean reads | 136093180 | 171712116 | 135462360 | 135660108 | 138631678 | 143511888 |
| Clean bases (Mb) | 13606.65 | 17165.08 | 13543.63 | 13551.37 | 13858.4 | 14345.03 |
| Clean data rate (%) | 99.96 | 99.93 | 99.96 | 99.86 | 99.95 | 99.93 |
| Clean read1 Q20 (%) | 98.36 | 98.65 | 98.33 | 98.48 | 98.48 | 98.46 |
| Clean read2 Q20 (%) | 97.01 | 97.93 | 96.82 | 97.12 | 97.22 | 97.22 |
| Clean read1 Q30 (%) | 92.85 | 94.05 | 92.75 | 93.39 | 93.36 | 93.28 |
| Clean read2 Q30 (%) | 89.78 | 92.59 | 89.25 | 90.25 | 90.42 | 90.46 |
| GC content (%) | 51.47 | 49.68 | 51.56 | 50.06 | 50.8 | 50.71 |

### Supplementary Table 2. Summary Statistics for SNPs

| **Sample ID** | **I-2** | **III-2** | **II-2** | **II-7** | **I-1** | **Average** |
| --- | --- | --- | --- | --- | --- | --- |
| Synonymous | 11027 | 11097 | 11020 | 10978 | 10969 | 11018 |
| Missense | 10423 | 10494 | 10440 | 10411 | 10440 | 10441 |
| Stopgain | 103 | 93 | 95 | 101 | 104 | 99 |
| Stoploss | 30 | 31 | 28 | 33 | 37 | 31 |
| Startloss | 21 | 23 | 19 | 21 | 22 | 21 |
| Splicing | 93 | 92 | 95 | 94 | 90 | 92 |
| Novel | 682 | 1466 | 718 | 2025 | 1041 | 1186 |
| Homozygous | 43005 | 49625 | 43977 | 46069 | 45684 | 45672 |
| Heterozygous | 51297 | 56587 | 52381 | 50093 | 51505 | 52372 |

### Supplementary Table 3. Summary Statistics for InDels

| **Sample ID** | **I-2** | **III-2** | **II-2** | **II-7** | **I-1** | **Average** |
| --- | --- | --- | --- | --- | --- | --- |
| Frameshift | 287 | 285 | 279 | 288 | 294 | 286 |
| Non-frameshift Insertion | 77 | 92 | 81 | 88 | 84 | 84 |
| Non-frameshift Deletion | 131 | 122 | 134 | 132 | 131 | 130 |
| Stoploss | 0 | 0 | 0 | 0 | 0 | 0 |
| Startloss | 1 | 0 | 1 | 1 | 0 | 0 |
| Splicing | 85 | 92 | 93 | 79 | 82 | 86 |
| Novel | 606 | 758 | 639 | 552 | 591 | 629 |
| Homozygous | 5761 | 7076 | 5901 | 6218 | 6184 | 6228 |
| Heterozygous | 7609 | 8383 | 7705 | 6831 | 7214 | 7548 |
